# Factors associated with the dietary patterns of Brazilian adolescents: analysis of the National Survey of School Health

**DOI:** 10.1016/j.jped.2024.09.006

**Published:** 2024-11-08

**Authors:** Alanna Gomes da Silva, Thales Philipe Rodrigues da Silva, Deborah Carvalho Malta

**Affiliations:** a Universidade Federal de Minas Gerais (UFMG), Escola de Enfermagem, Departamento de Enfermagem Materno Infantil e Saúde Pública, Belo Horizonte, MG, Brazil; b Universidade Federal de São Paulo (UNIFESP), Escola Paulista de Enfermagem, Departamento de Enfermagem na Saúde da Mulher, São Paulo, SP, Brazil

**Keywords:** Adolescent, Feeding behavior, Food consumption, Health promotion, Health surveys, Brazil

## Abstract

**Objectives:**

To identify the dietary patterns of Brazilian adolescents and to verify their associated factors.

**Methods:**

Cross-sectional study with data from the 2019 National Survey of School Health. Students aged 13 to 17 participated in the survey, totaling a sample of 125,123 adolescents. The variables were divided into two groups: healthy and unhealthy diet. Principal component analysis was used to identify the dietary patterns. To evaluate the association, logistic regression was used, estimated by the Odds Ratio, with the respective 95 % confidence intervals.

**Results:**

Two main components were identified: first related to regular consumption of fruits, vegetables, and beans; the second related to non-regular consumption of sweet treats, soft drinks, and fast food. The highest likelihood of regularly consuming the first was observed among adolescents residing in the Central-West and Southeast regions, with higher maternal education, who abstained from alcohol, engaged in physical activity, were not sedentary, ate breakfast, had lunch or dinner with their parents, refrained from eating while engaging in other activities, and participated in school meal programs. The lowest likelihood of not regularly consuming the second was found among male adolescents aged 16 to 17, attending public schools, who abstained from alcohol, and were not sedentary.

**Conclusions:**

This study identified two dietary patterns, both linked to socioeconomic factors and healthy lifestyle habits. Recognizing these patterns among adolescents enables health surveillance efforts aimed at reducing diseases and health problems.

## Introduction

Nutritional transition in Brazil has led to significant changes in the population's dietary patterns over the past decades, characterized by an increase in the consumption of ultra-processed foods, high levels of sugars, fats, and sodium, and a reduction in the intake of natural or minimally processed foods.^1^ The Brazilian nutritional transition has been marked by a decline in malnutrition and a rise in overweight and obesity rates.^2^

The change in the dietary profile of the population has not only occurred in adults but also among adolescents. A study with data from the 2019 National Survey of School Health (PeNSE) showed that the consumption of ultra-processed foods was 97.3 % and there was a reduction in the consumption of fresh fruits and vegetables from 2015 to 2019.^3^ In 2022, over 4.4 million adolescents aged 10 to 19 were monitored through the Ministry of Health's Food and Nutrition Surveillance System. Of these, nearly 1.4 million were diagnosed with overweight, obesity, or severe obesity.^4^

This scenario is worrisome because unhealthy dietary patterns can lead to both nutritional deficiencies and also excesses, which may affect the growth process inherent in puberty and contribute to the increase in Food and Nutritional Insecurity, overweight and obesity, and the development of non-communicable diseases (NCDs).^2^^,^^5^

Adolescence represents a critical period for the establishment of dietary habits that may persist into adulthood and significantly impact health. In this context, identifying the dietary patterns of Brazilian adolescents is essential, as it enables a deeper understanding of the eating behaviors within this age group and helps to identify the factors associated with these habits. Food consumption is influenced by multiple determinants, including food availability and access, as well as individual, family, social, school, contextual, and health-related factors.^6^ Furthermore, it provides a more integrated and comprehensive view of dietary behavior compared to those that assess food consumption in isolation, offering stronger evidence to support food and nutrition surveillance actions and to inform public health policies aimed at promoting access to healthy foods for the entire population, particularly the most vulnerable groups.^7^

This study aimed to identify the dietary patterns of Brazilian adolescents and to verify their associated factors.

## Methods

### Study design

This is an epidemiological study, with a cross-sectional design carried out with data from the National Survey of School Health (PeNSE), carried out from April 9 to September 30, 2019.

### Setting

PeNSE is a survey conducted with adolescent students, conducted by the Brazilian Institute of Geography and Statistics (IBGE), in partnership with the Ministry of Health and support from the Ministry of Education. The Research is part of the Surveillance of Risk and Protective Factors for NCDs in Brazil and addresses various aspects of adolescents’ lives, such as habits, care, risk, and protective factors for their health.^8^

### Data collection instrument

The final version of the data collection instruments for PeNSE 2019 was established after a process of structuring and testing. Initially, the topics and questions to be investigated were evaluated in collaboration with the Health Surveillance Secretariat of the Ministry of Health. Subsequently, this preliminary version of the instruments underwent two stages of testing to assess the completion time of the questionnaires and the students's understanding of the wording of the pre-selected questions.^8^

Data collection was carried out using the Mobile Collection Device (MCD), which corresponds to a smartphone containing the structured questionnaire of the research. Each instrument had specific instructions for completion, with the Student Questionnaire being self-administered.

### Participants

Adolescent students aged 13 to 17, enrolled and regularly attending the 7th to 9th grades of Elementary School and the 1st to 3rd grades of High School, including technical courses with integrated high school and normal/teaching courses, from all shifts, in public and private schools in Brazil participated in the research.

In 2019, data were collected from 4242 schools, 6612 classes, with 189,857 students enrolled and 183,264 students in attendance, of which 159,245 were valid questionnaires. In other words, the questionnaires of students who indicated their consent to participate in the research in the MCD and provided their gender and age were included, resulting in a total of 125,123 being analyzed. This was due to the exclusion of those younger than 13 or older than 18 years of age, as well as those with missing responses.

Additional information on the methodological and operational aspects is available in the survey publication.^8^

### Variables’ description

#### Dependent variable

These variables were subsequently used in Principal Component Analysis (PCA) to generate the dietary patterns. Thus, these variables were categorized as belonging to the 1st and 2nd tertile and to the 3rd tertile (Supplementary Material 1).

#### Independent variables

The independent variables were categorized as sociodemographic: gender (male and female); age group (13 to 15; 16 and 17); skin color (white, black, brown, and other – yellow and indigenous); region (North, Northeast, Southeast, South, and Central-West); administrative dependency of the school (private and public); maternal schooling (No schooling and incomplete elementary school, Complete elementary school and incomplete high school, Complete high school and Incomplete higher education, and Complete higher education); and Daily habits: Current use of cigarettes (Yes, No); Current consumption of alcoholic beverages (Yes, No); Physical activity (≥ 300 min per week and ≤ 300 min per week); Sedentary behavior (≥ 3 h sitting per day and ≤ 3 h sitting per day); Eating breakfast five or more days a week (No, Yes); Having lunch or dinner with parents or guardians five or more days a week (No, Yes); Eating meals at the same time as other activities, such as watching TV, using the computer or cell phone (Yes, No); Consuming meals offered by the school (No, Yes).

### Statistical analysis

The descriptive analysis included the calculation of prevalence and the respective 95% confidence intervals (95 %CI).

To identify patterns of dietary pattern, principal component analysis (PCA) was performed, and the variables included were: Regular consumption of fruits, vegetables, and beans; Regular consumption of sweet treats, soft drinks, and fast food.

The Kaiser-Mayer-Olkin (KMO) coefficient was used to measure the adequacy of the PCA, with values from 0.5 to 1.0 being considered. The formation of the components was confirmed by the “Bertlett's Test of Sphericity” with a result ≤0.05 and by the “Scree Plot.”

The component structure was obtained from the variables with factor loadings greater than 0.3 or less than −0.3. Subsequently, the scores of the patterns generated were extracted and a binary variable was created based on the tertile of these scores.

After identifying the scores generated, a binary variable was created based on the tertiles generated, grouping the 1st and 2nd tertiles due to their similar characteristics. Therefore, adolescents were categorized into two groups: those belonging to the 1st and 2nd tertiles and those in the 3rd tertile, resulting in a dichotomous variable.

Logistic regression was used to assess associations, with the Odds Ratio (OR) and respective 95% CI estimating the magnitude of these associations. The Backward method was employed for the construction of the multivariate regression model, where variables related to a level of statistical significance lower than 20% in the bivariate analysis were included and subsequently removed one by one. The multivariate analysis was conducted to adjust for potential confounding factors, including gender, age, and the administrative dependency of the school. These factors were considered based on the literature and their possible influences on the outcome variable. Statistical significance was set at a p-value of <0.05.

The analyses were performed with the Stata version 14.2 software, using the survey module, which considers post-stratification weights.

Due to PeNSE's complex sampling design and sample losses, post-stratification weights were considered in all the analyses.

### Ethical considerations

PeNSE was approved by the National Research Ethics Committee for Human Beings of the Brazilian Ministry of Health (Opinion No No 3.249.268, of April 8, 2019).

## Results

Considering the values adopted, the first and second components were extracted, which showed a variance of 46 %. The first component was characterized by regular consumption of healthy foods (fruits, vegetables, and beans). The second component was characterized by a mixed pattern (non-regular consumption of sweet treats, soft drinks, and fast food). The analysis achieved a satisfactory KMO (Supplementary Material 2).

The characteristics of adolescents belonging to pattern 1 are described in Tables 1 and 2 provides a detailed description of the characteristics of adolescents associated with pattern 2.

**Table 1:** Characteristics of adolescents who belong to Pattern 1 and univariate logistic regression. National Survey of School Health. Brazil, 2019.

	Pattern 1 (regular consumption of fruits, vegetables, and beans)		
Variables	% (95% CI)	*P*-value	Odds Ratio (95% CI)
*Gender*		<0.001	
Female	29.06 (28.17–29.96)		⁎
Male	33.84 (32.97–34.71)		1.24 (1.17–1.32)
*Age*		<0.001	
13 to 15	32.9 (32.14–33.67)		⁎
16 and 17	28.68 (27.7–29.69)		0.82 (0.77–0.87)
*Skin color*		0.036	
White	32.35 (31.49–33.23)		⁎
Black	30.44 (29.02–31.89)		0.91 (0.84–0.98)
Mixed	31.1 (30.24–31.97)		0.94 (0.89–0.99)
Others	30.12 (28.08–32.24)		0.90 (0.81–1.00)
*Region*		<0.001	
North	23.33 (21.81–24.91)		⁎
Northeast	27.83 (26.81–28.88)		1.26 (1.14–1.40)
Southeast	35.26 (34.15–36.39)		1.79 (1.62–1.97)
South	31.17 (29.76–32.62)		1.48 (1.33–1.66)
Central-West	36.61 (35.32–37.93)		1.89 (1.71–2.10)
*Administrative dependency*		<0. 001	
Private	34.15 (33.22–35.09)		⁎
Public	30.95 (30.25–31.65)		0.86 (0.81–0.91)
*Maternal schooling*		<0.001	
Illiterate and incomplete elementary school	27.97 (26.76–29.22)		⁎
Incomplete elementary and high school	30.94 (29.39–32.53)		1.15 (1.05–1.25)
Incomplete high school and higher education	32.02 (31.0 - 33.07)		1.21 (1.12–1.31)
Higher education and more	37.39 (36.27–38.53)		1.53 (1.42–1.66)
*Cigarettes*		<0.001	
Yes	26.62 (24.5–28.85)		⁎
No	31.77 (31.14–32.4)		1.28 (1.14–1.44)
*Alcoholic beverages*		<0.001	
Yes	28.38 (27.28–29.51)		⁎
No	32.62 (31.93–33.32)		1.22 (1.14–1.29)
*Physical activity*		<0.001	
< 300 min	27.08 (26.4–27.77)		⁎
> 300 min	42.05 (40.92–43.19)		1.95 (1.84–2.06)
*Sedentary*		<0.001	
> 3 h/day	28.81 (28.06–29.58)		⁎
< 3 h/day	34.48 (33.55–35.42)		1.29 (1.23–1.37)
*Eating breakfast*		<0.001	
No	24.91 (24.11–25.72)		⁎
Yes	35.88 (35.12–36.65)		1.68 (1.60–1.77)
*Having lunch or dinner with parents*		<0.001	
No	23.78 (22.85–24.74)		⁎
Yes	34.86 (34.12–35.62)		1.71 (1.61–1.82)
*Eating meals with other activities*		<0.001	
Yes	29.26 (28.56–29.98)		⁎
No	36.96 (35.66–38.27)		1.41 (1.32–1.51)
*School meal*		<0.001	
No	30.5 (29.74–31.27)		⁎
Yes	32.83 (31.81–33.87)		1.11 (1.04–1.18)

⁎ Reference group.

**Table 2:** Characteristics of adolescents who belong to Pattern 2 and univariate logistic regression. National Survey of School Health. Brazil, 2019.

	Pattern 2 (non-regular consumption of sweet treats, soft drinks, and fast food)		
Variables	% (95% CI)	*P*-value	Odds ratio (95% CI)
*Gender*		<0.001	
Female	32.55 (31.73–33.38 %)		⁎
Male	36.43 (35.62–37.24)		1.18 (1.13–1.24)
*Age*		<0.001	
13 to 15	33.61 (32.82–34.41)		⁎
16 and 17	36.02 (34.95–37.1)		1.11 (1.04–1.18)
*Skin color*		<0.001	
White	31.77 (30.81–32.76)		⁎
Black	36.1 (34.42–37.81)		1.21 (1.11–1.31)
Mixed	35.89 (34.96–36.84)		1.20 (1.12–1.28)
Others	35.83 (33.83–37.88)		1.19 (1.08–1.31)
*Region*		<0.001	
North	40.44 (38.7–42.2)		⁎
Northeast	41.25 (40.11–42.4)		1.03 (0.94–1.12)
Southeast	30.2 (29.13–31.28)		0.63 (0.58–0.69)
South	31.11 (29.61–32.65)		0.66 (0.60–0.73)
Central-West	28.89 (27.73–30.09)		0.59 (0.54–0.65)
*Administrative dependency*		<0.001	
Private	26.7 (26.07–27.33)		⁎
Public	35.77 (35.05–36.5)		1.52 (1.46–1.60)
*Maternal schooling*		<0.001	
Illiterate and incomplete elementary school	40.51 (39.2–41.83)		⁎
Incomplete elementary and high school	33.67 (31.97–35.41)		0.74 (0.68–0.81)
Incomplete high school and higher education	32.3 (31.24–33.37)		0.70 (0.65–0.75)
Higher education and more	25.99 (24.93–27.07)		0.51 (0.47–0.55)
*Cigarettes*		<0.001	
Yes	30.89 (29.12–32.72)		⁎
No	34.71 (34.07–35.36)		1.18 (1.09–1.29)
*Alcoholic beverages*		<0.001	
Yes	29.85 (28.87–30.85)		⁎
No	36.25 (35.51–36.98)		1.33 (1.26–1.40)
*Physical activity*		<0.001	
< 300 min	37.42 (36.64–38.21)		⁎
> 300 min	27.2 (26.27–28.15)		0.62 (0.58–0.66)
*Sedentary*		<0.001	
> 3 h/day	31.6 (30.87–32.35)		⁎
< 3 h/day	37.77 (36.85–38.7)		1.31 (1.25–1.37)
*Eating breakfast*		0.09	
No	35.06 (34.12–36.01)		⁎
Yes	34.04 (33.27–34.82)		0.95 (0.90–1.00)
*Having lunch or dinner with parents*		<0.001	
No	36.15 (35.14–37.18)		⁎
Yes	33.7 (32.95–34.45)		0.89 (0.85–0.94)
*Eating meals with other activities*		0.01	
Yes	33.94 (33.28–34.61)		⁎
No	35.76 (34.52–37.03)		1.08 (1.02–1.14)
*School meal*		0.012	
No	33.85 (33.14–34.58)		⁎
Yes	35.4 (34.36–36.47)		1.07 (1.01–1.13)

⁎ Reference group.

The final multivariate model for Pattern 1, is outlined in Table 3. The more likely of regularly consume fruits, vegetables, and beans were entre adolescents residing in all regions, particularly the Central-West (OR = 2.21; 95% CI: 1.95–2.51), with higher maternal schooling (OR = 1.48; 95% CI: 1.36–1.60), who did not consume alcoholic beverages (OR = 1.09; 95% CI: 1.01–1.17), who practiced physical activity (OR = 1.88; 95% CI: 1.77–2.00), were not sedentary (OR = 1.20; 95% CI: 1.13–1.28), who ate breakfast (OR = 1.64; 95% CI: 1.54–1.74), had lunch or dinner with their parents (OR = 1.55; 95% CI: 1.45–1.66), did not eat with other activities (OR = 1.31; 95% CI: 1.22–1.41), and ate school meals (OR = 1.08; 95% CI: 1.01–1.16).

**Table 3:** Multivariate model for Pattern 1 - Regular consumption of fruits, vegetables, and beans. National Survey of School Health. Brazil, 2019.

Variables	Odds ratio %(95% CI)
*Age*	
13 to 15	⁎
16 and 17	0.89 (0.84–0.96)
*Region*	
North	⁎
Northeast	1.39 (1.23–1.58)
Southeast	2.18 (1.93–2.45)
South	1.67 (1.47–1.90)
Central-West	2.21 (1.95–2.51)
*Maternal schooling*	
Illiterate and incomplete elementary school	⁎
Complete elementary and incomplete high school	1.14 (1.04–1.24)
Complete high school and incomplete higher education	1.21 (1.12–1.31)
Complete higher education and more	1.48 (1.36–1.60)
*Alcoholic beverages*	
Yes	⁎
No	1.09 (1.01–1.17)
*Physical activity*	
< 300 min	⁎
> 300 min	1.88 (1.77–2.00)
*Sedentary behavior*	
> 3 h/day	⁎
< 3 h/day	1.20 (1.13–1.28)
*Eating breakfast*	
No	
Yes	1.64 (1.54–1.74)
*Having lunch or dinner with parents*	
No	
Yes	1.55 (1.45–1.66)
*Eating meals with other activities*	
Yes	⁎
No	1.31 (1.22–1.41)
*School meal*	
No	
Yes	1.08 (1.01–1.16)

⁎ Reference group.

Table 4 presents the multivariate model for Pattern 2. The likelihood of non-regular consumption of sweets, soft drinks, and fast food was higher among male adolescents (OR = 1.32; 95% CI: 1.25–1.39), aged 16 and 17 (OR = 1.16; 95% CI: 1.08–1.23), from public schools (OR = 1.24; 95% CI: 1.17–1.31), who did not consume alcoholic beverages (OR = 1.28 95 %CI: 1.21–1.36), who were not sedentary (OR = 1.21 95% CI: 1.14–1.29).

**Table 4:** Multivariate model for Pattern 2 - Non-regular consumption of sweet treats, soft drinks, and fast food. National Survey of School Health. Brazil, 2019.

Variables	Odds ratio %(95% CI)
*Gender*	
Female	⁎
Male	1.32 (1.25–1.39)
*Age*	
13 to 15	⁎
16 and 17	1.16 (1.08–1.23)
*Region*	
North	⁎
Northeast	0.99 (0.90–1.08)
Southeast	0.64 (0.58–0.70)
South	0.69 (0.62–0.77)
Central-West	0.61 (0.55–0.67)
*Administrative dependency*	
Private	⁎
Public	1.24 (1.17–1.31)
*Maternal schooling*	
Illiterate and incomplete elementary school	⁎
Complete elementary and incomplete high school	0.79 (0.72–0.86)
Complete high school and incomplete higher education	0.78 (0.72–0.84)
Complete higher education and more	0.61 (0.55–0.66)
*Alcoholic beverages*	
Yes	⁎
No	1.28 (1.21–1.36)
*Physical activity*	
< 300 min	⁎
> 300 min	0.60 (0.56–0.65)
*Sedentary behavior*	
> 3 h/day	⁎
< 3 h/day	1.21 (1.14–1.29)
*Eating breakfast*	
No	⁎
Yes	0.81 (0.76–0.87)
*Having lunch or dinner with parents*	
No	⁎
Yes	0.82 (0.78–0.87)

⁎ Reference group.

## Discussion

From the PCA it was possible to verify two patterns, the first related to a healthy food consumption pattern by the regular consumption of fruits, vegetables, and beans and the second to a mixed pattern since it cannot be considered a healthy dietary pattern just not to regularly consume sweet treats, soft drinks, and fast-food.

Fruits, vegetables, and greens are essential for a healthy dietary pattern and act as protective factors against NCDs).^9^ Beans, a culturally significant food in Brazilian meals, serve as an important indicator of healthy eating in the country.^10^ However, the consumption of these foods is determined by various factors in different contexts and can be influenced by socioeconomic and demographic characteristics.^11^ Brazil has a vast territorial extent, and differences in climate, culture, and economic activities are reflected in the diverse dietary habits of its population, as well as in the supply, availability, and access to these foods. The Southeast region accounts for the majority of the volume of fruits and vegetables traded, while the North has the lowest percentage, which affects the price and accessibility for the end consumer. Individuals living in economically disadvantaged regions have lower consumption of healthy foods, a consequence of the distribution, availability, and prices of these foods, as well as lower income to purchase them.^12^

In this context, parental education levels are factors that influence the type of dietary patterns adolescents adopt,^7^ as individuals with higher education levels tend to have better income and employment, which facilitates the consumption of healthier foods. Higher education also possibly leads to greater knowledge about nutrition and dietary recommendations for food choices, as well as better access to healthy food options.^13^

These data show the disparities between dietary patterns and socioeconomic levels, which reflects the need for intersectoral public policies that encompass everything from food production to consumption. There is still a need for progress in market regulation strategies that provide opportunities for the production and distribution of fruits and vegetables, in establishing policies to tackle the determinants of food insecurity, reducing food prices, and in strengthening income transfer programs and policies to strengthen familiar agriculture.

Not consuming alcoholic beverages, physical activity practice, and avoiding a sedentary lifestyle are also protective factors against NCDs, as they can reduce the risk of cardiovascular diseases, diabetes, and various types of cancer, in addition to helping with weight control and improving the quality of life and mental health.^14^ For both patterns, there was an association with healthy lifestyle habits. Health behaviors tend to occur simultaneously, due to the synergistic relationship. More active individuals are motivated to eat healthier.^15^^,^^16^ On the other hand, the coexistence of health risk factors is also a reality among adolescents.^17^^,^^18^ Therefore, understanding and monitoring lifestyle habits in adolescence is essential for developing health promotion actions aimed at multiple health behaviors and consequently reducing the burden of disease in adult life.

Breakfast is often described as the most important meal of the day. The nutritional quality and regularity of breakfast consumption are associated with better physical, mental, and academic performance, as well as better engagement in physical activity, quality of life, and overall health.^19^ Children and adolescents who regularly eat breakfast have a higher intake of several nutrients, especially vitamins and minerals, compared to those who skip breakfast.^20^ However, despite these benefits, the prevalence of children and adolescents skipping breakfast is increasing, particularly among those with lower socioeconomic status.^20^ The habit of skipping breakfast is also associated with higher consumption of ultra-processed foods and other obesogenic behaviors.^17^ Promoting the habit of eating breakfast among young people can be facilitated by political and educational measures.

Eating meals with parents or guardians provides benefits for adolescents, such as promoting healthy eating habits, increasing the consumption of fruits, vegetables, whole grains, and beans, and reducing the intake of ultra-processed foods. Additionally, it contributes to improved psychosocial well-being and academic performance, while providing emotional support, more dialogue, and greater contact between parents or guardians and adolescents.^17^^,^^21^, ^22^, ^23^ These benefits may be related to greater parental control over meal routines, which allows them to monitor food intake and influence healthy food choices, as well as promote regular mealtimes.^21^, ^22^, ^23^ However, it is important to consider that there are differences in family environment characteristics and meals, such as the diversity, quality, and quantity of food consumed, family interpersonal relationships, meal planning, eating while watching TV, parental education, family income, and parent's employment status, all of which also influence dietary patterns and lifestyle.^21^^,^^23^

Exposure to screens is considered a sedentary behavior and causes distractions that interfere with physiological signals of hunger and satiety, leading to inappropriate food choices such as the consumption of ultra-processed foods and contributing to overweight in the early stages of life. One study showed that most adolescents reported consuming snacks in front of screens sometimes, and approximately 40.0 % almost always or always adopted this practice.^24^

Regarding school meals, the Brazilian National School Feeding Program ensures the provision of adequate and healthy food, ensuring food and nutritional security, along with health education actions for public school students, which encourages the formation of healthy dietary pattern.^25^ The program aims to foster healthy eating habits through food and nutrition education initiatives and by offering free meals that meet all the nutritional needs of public school students. The menus are planned based on fresh, minimally processed foods, with a ban on sugary foods and restrictions on ultra-processed items high in sodium or saturated fats.^25^ For this reason, adolescents who eat school meals are more likely to consume fruits, vegetables, and beans. This highlights the importance of maintaining and investing in health promotion programs, as they positively impact quality of life and improve students’ dietary patterns.

In this regard, adolescents attending public schools were more likely to exhibit non-regular consumption of sweet treats, soft drinks, and fast food. This may be related to the school meals program, which promotes healthy eating as mentioned earlier, but also to economic factors that influence the purchase of these foods. Generally, low- or middle-income families are more likely to enroll their children in public schools, while higher-income families tend to opt for private schools.^26^ A study based on the PeNSE found that adolescents with a higher socioeconomic status had a higher consumption of ultra-processed foods,^13^ because unlike high-income countries, where these foods are cheaper and easily accessible, in Brazil they are still more expensive, especially in local shops compared to supermarkets.^27^

Regarding eating habits by gender, national studies show differences in behavior when comparing boys and girls. Male adolescents exhibited more frequent consumption of soft drinks,^3^ higher average intake of energy and sodium,^28^ and greater consumption of fruits, vegetables, and beans.^3^ In contrast, girls consumed more snacks in front of screens^24^ and sweet treats.^3^ The consumption of ultra-processed foods and fresh fruits or fruit salads was similar between the genders.^3^ These differences may be influenced by various factors, such as social and cultural pressures, concerns about physical appearance, and the dietary patterns of their families and social environment.

Adolescents' eating habits vary significantly according to age group. The transition from childhood to adolescence brings physiological changes and increased influence from the social environment, leading to shifts in dietary patterns.^29^ Younger adolescents tend to have diets more guided by their parents, while older adolescents are influenced by external factors, such as friends and social media, and experience greater independence in decision-making. Additionally, exposure to stressful situations and social pressures in late adolescence can lead to an increase in the consumption of unhealthy foods and lifestyles.^7^^,^^18^^,^^29^ The results of this study showed that older adolescents are more likely to avoid unhealthy foods; however, this does not imply that they do not consume such foods. Therefore, it is essential to further explore the factors influencing eating behavior throughout adolescence, including the transition to adulthood, in order to assess changes in diet and the influence of family, school, work, and social environments.

As a limitation of the study, it should be considered that the 2019 PeNSE questionnaire omitted several questions regarding the regular consumption of healthy and unhealthy foods over the past seven days, which limited the selection of additional dependent variables. However, it was possible to select those considered by the literature as markers of a healthy and protective diet against NCDs.

This study identified two primary dietary patterns. The first, is classified as a healthy pattern due to the consumption of fruit, vegetables, and beans. The second, a mixed pattern, was evidenced by the irregular consumption of sweet treats, soft drinks, and fast foods. Both patterns were associated with socioeconomic variables and healthy lifestyle habits. To monitoring the health behavior of adolescents remains crucial to supporting public surveillance, strengthening social and school-based policies, reducing inequalities, and improving access to healthy food and health promotion services among adolescents.

## Funding sources

10.13039/501100004901Fundação de Amparo à Pesquisa do Estado de Minas Gerais (FAPEMIG). Programa de Apoio à Fixação de Jovens Doutores no Brasil (Processo: BPD-00893–22).

## Conflicts of interest

The authors declare no conflicts of interest.

## Appendix Supplementary materials

Image, application 1
