# Supplementary material for: Factors associated with the dietary patterns of Brazilian adolescents: analysis of the National Survey of School Health
Source: J Pediatr (Rio J). 2024 Nov 8;101(2):247–54. doi: 10.1016/j.jped.2024.09.006 (PMC11889679; doi:10.1016/j.jped.2024.09.006)
Supplement: Supplementary file 1 [file mmc1.docx]

**JPED-D-24-00215_ Supplementary Material**

**Supplementary Material 1** Study dependent variables. National School Health Survey, Brazil, 2019.

| **Variables** | **Survey question** | **Definition adopted** |
| --- | --- | --- |
| Regular consumption of fruits | DURING THE PAST 7 DAYS, on how many days did you consume fresh fruit or fruit salad? | Consumption of fruit on five or more days was considered regular. |
| Regular consumption of vegetables | DURING THE PAST 7 DAYS, on how many days did you consume at least one type of vegetable other than potatoes or cassava? | Consumption of vegetables on five or more days was considered regular. |
| Regular consumption of beans | DURING THE PAST 7 DAYS, on how many days did you consume beans? | Consumption of beans on five or more days was considered regular. |
| Regular consumption of sweets | DURING THE PAST 7 DAYS, on how many days did you consume sweet treats, such as candies, confectionery, chocolates, chewing gum, bonbons, lollipops, and others? | Consumption of sweets on five or more days was considered regular. |
| Regular consumption of soft drinks | DURING THE PAST 7 DAYS, on how many days did you consume soft drinks? | Consumption of soft drinks on five or more days was considered regular. |
| Food consumption at snack bars | IN THE LAST 7 DAYS, on how many days did you consume at snack bars, hot dog stands, pizzerias, fast food, etc.? | Eating at snack bars on five or more days was considered regular. |

**Supplementary Material 2** Factor loadings of patterns extracted from the principal component analysis.

| **Variables** | **Pattern 1** | **Pattern 2** | **Kaiser-Mayer-Olkin** |
| --- | --- | --- | --- |
| Consumption of fruits | 0.6076 | -0.1901 | 0.5330 |
| Consumption of vegetables | 0.6397 | -0.1268 | 0.5331 |
| Consumption of beans | 0.4114 | -0.0418 | 0.6460 |
| Consumption of sweets | 0.1328 | 0.5479 | 0.5740 |
| Consumption of soft drinks | 0.1718 | 0.5940 | 0.5555 |
| Eating at snack bars | 0.0720 | 0.5413 | 0.5875 |
| Eigenvalue | 1.4183 | 1.3625 |  |
| Explained variance | 0.2364 | 0.2271. |  |
| Accumulated explained variance | 0.2364 | 0.4635 |  |
| Overall |  |  | 0.5551 |
